# Overinfection by *Paracoccidioides brasiliensis* in Gouty Crystal Arthritis

**DOI:** 10.1155/2012/128103

**Published:** 2012-11-10

**Authors:** F. Bonilla-Abadía, J. D. Vélez, L. C. Zárate-Correa, E. Carrascal, N. Guarín, C. R. Castañeda-Ramírez, C. A. Cañas

**Affiliations:** ^1^Rheumatology Unit, Fundación Valle del Lili, ICESI University, Cali, Colombia; ^2^Infectious Diseases Unit, Fundación Valle del Lili, Cali, Colombia; ^3^Internal Medicine Unit, Fundación Valle del Lili-CES University, Cali, Colombia; ^4^Pathology Unit, Fundación Valle del Lili-ICESI University and Faculty of Health Sciences, Universidad del Valle, Cali, Colombia; ^5^Pathology Unit, Fundación Valle del Lili-ICESI University, Cali, Colombia; ^6^Medical Microbiology and Infectious Diseases Unit, Fundación Valle del Lili-ICESI University and Universidad del Valle, Cali, Colombia

## Abstract

Paracoccidioidomycosis is an endemic South American systemic mycosis caused by the dimorphic fungus *Paracoccidioides brasiliensis* (*P. brasiliensis*). The main clinical form of disease is pulmonary, but all organs may be involved. We report a case of overinfection by *P. brasiliensis* in chronic gouty arthritis affecting the proximal phalanx of the right hallux. The patient required proximal amputation and long-term antifungal therapy.

## 1. Introduction

Paracoccidioidomycosis is a systemic mycosis endemic in South America and caused by the dimorphic fungus *Paracoccidioides brasiliensis (P. brasiliensis)* [1]. The infection occurs by inhalation of environmental airborne or through skin and mucosal lesions. The main clinical form of disease is pulmonary, but all organs may be involved [1, 2]. We report a case of overinfection by *P. brasiliensis* in chronic gouty arthritis affecting the proximal phalanx of the right hallux, confirmed by culture, histophathological specimen, and serological agar gel immunodiffusion.

## 2. Case Report

An 82-year-old male was admitted by one-month history of swelling and small pustules that progressed to ulcers with purulent drainage in his right hallux. Medical history includes recurrent gout attacks since 8 years ago, hypertension, diabetes mellitus, hypothyroidism, and chronic kidney failure in medical management, aortic valve replacement, and myocardial revascularization. He lived in Buga, Colombia, and worked in agriculture but had lived in Chiclayo, Peru, for six years; both are endemic areas of *P. brasiliensis* [3, 4].

**Figure 1:**
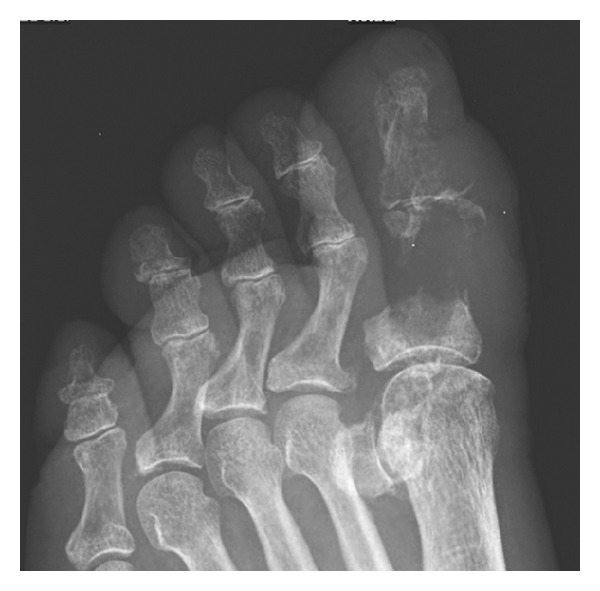
X-ray of right foot showing erosive and lytic lesion with cortical disruption in the proximal phalanx of the right hallux associated with soft tissue swelling.

**Figure 2:**
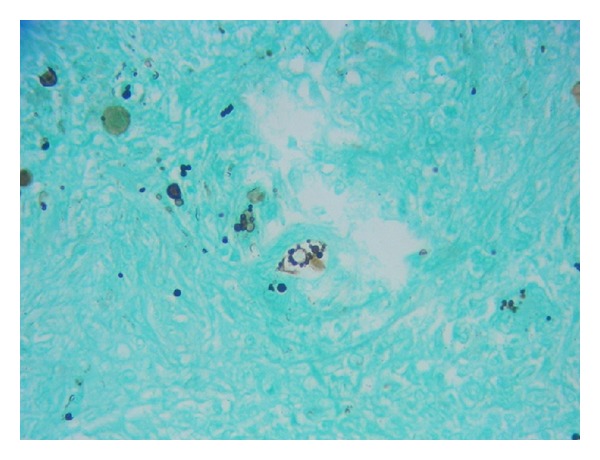
Multiple budding “pilot wheel” morphology of *P. brasiliensis* (methenamine silver stain).

At exam, the hallux was edematous with limited range of motion and mild pain, erythema, and two ulcers of 2 cm on the lateral and medial aspect of the foot. The patient was afebrile, and findings on general medical and pulmonary tests were unremarkable. A plain radiograph of the foot revealed a lytic lesion with cortical disruption in the proximal phalanx of the right hallux associated with soft tissue swelling (Figure 1). The chest radiograph was normal. Laboratory studies showed a PCR of 4.47 mg/dL (0–0.5 mg/dL), BUN 42 mg/dL (8–23 mg/dL), serum creatinine of 1.7 mg/dL, TSH level of 6.4 mIU/L, albumin 2.6 g/dL, white blood cell count of 8 × 10^3^/uL with a normal differential, hemoglobin of 11.2 g/dL, hematocrit of 35%, and platelets of 390.000 K/uL. HIV test was negative. Blood and urine cultures were reported as negative. An amputation of the hallux was done. The microbiology laboratory received the sample of drainage right hallux for bacteriological study, to which Gram stain was initially performed and fungal structures were observed like a large, round, fairly thick-walled cells with single and multiple buddings. Direct examination, with KOH 10%, observed unique spherical yeast budding and also observed crystal needle-shaped structures, which observed polarized light filters with characteristic of monosodium urate crystal. At 35°–37°C on liquid medium, was obtained multiple budding “pilot wheel” morphology of *P. brasiliensis*. Histopathological study and bone cultures for aerobic, anaerobic, and fungal growth were done. The pathology report was consistent with osteomyelitis. Histologic examination revealed multiplebudding cells consistent with *P. brasiliensis* (Figure 2) and confirmed by isolation of the fungus in cultures and serological complementary diagnosis with agar gel immunodiffusion.

A diagnosis of a secondary infection by *P. brasiliensis* in a chronic gouty arthritis (right hallux) was made, and therapy with itraconazole was initiated without evidence of adverse effect. The patient's clinical course was satisfactory. 

## 3. Discussion

Paracoccidioidomycosis is a systemic granulomatous disease caused by the dimorphic fungus *P. brasiliensis*. It is usually found in South America, and it is considered an endemic disease and a public health problem especially in countries as Brazil, Venezuela, Colombia, Ecuador, and Argentina [5, 6]. Paracoccidioidomycosis can be classified in acute/subacute and chronic forms [5]. The acute/subacute form accounts for 3%–5% of all cases and is frequently seen in children and adolescents, without gender differences. It is characterized by a fast progression, lymphadenopathy, gastrointestinal manifestations, hepatosplenomegaly, osteoarticular involvement, and cutaneous lesions. The chronic form is most commonly seen in adults, predominantly in males between the third and sixth decade [7] and can be unifocal (affecting only the lungs) or multifocal (affecting lungs, mucosa, and skin) [5, 6]. This infection can be successfully treated with oral antifungals like itraconazole, used daily for a long period [5].

Paracoccidioidomycosis is more common in males, especially in the third and fourth decades of life [8, 9]. *P. brasiliensis* extends to the lungs by respiratory route and can infect persons exposed to the environment where the fungus survives in the saprophytic form. The host may control the primary infection, but dissemination might occur to other tissues by haematogenous and lymphatic routes. 

The osteoarticular involvement in paracoccidioidomycosis can be the first or the only system involved and has a variable prevalence [10, 11]. Bone lesions have a prevalence range in the literature from 2% to 30% [12, 13], and articular and muscular lesions are very uncommon [10, 14]. A recent study of septic arthritis in monoarthritis crystals was published, but there was no evidence of fungal infection within the results [15]. 

In the disseminated form of paracoccidioidomycosis, osteoarticular involvement could reach up to 20% of cases [8], and some reports have found only bone involvement in the acute clinical form [16, 17]. These severe forms of this disease may be complicated to hypercalcemia and hemophagocytic syndromes [18]. 

The rates of osteoarticular involvement by *P. brasiliensis* are completely different between patients with acute and chronic infections, occurring almost exclusively in the acute/subacute clinical form [16]. 

The bone lesions studied with conventional radiography have been described as sharply defined osteolytic lesions without marginal sclerosis and without periosteal reaction [13, 15], and any bone can be involved [8, 9, 19]. 

We report a very uncommon case of septic arthritis by paracoccidioidomycosis on monosodium urate crystal arthritis with a progressive and poor evolution associated with the development of an osteomyelitic lesion, requiring proximal amputation and long-term antifungal therapy. Despite absence of clinical signs and symptoms of lung involvement, a CT scan was not performed and lung disease may not be completely roled out.

Amphotericin was contraindicated in this patient because of potential renal toxicity, and the treatment with itraconazole was closely watched by their possible cardiovascular adverse effects [20] and negative inotropic action [21]. Therefore, posaconazole could become an important antifungal option.
